# The ISG15-specific protease USP18 regulates stability of PTEN

**DOI:** 10.18632/oncotarget.13914

**Published:** 2016-12-12

**Authors:** Lisa Maria Mustachio, Masanori Kawakami, Yun Lu, Jaime Rodriguez-Canales, Barbara Mino, Carmen Behrens, Ignacio Wistuba, Neus Bota-Rabassedas, Jun Yu, J. Jack Lee, Jason Roszik, Lin Zheng, Xi Liu, Sarah J. Freemantle, Ethan Dmitrovsky

**Affiliations:** ^1^ Department of Pharmacology and Toxicology, Geisel School of Medicine at Dartmouth, Hanover, NH, USA; ^2^ Department of Thoracic/Head and Neck Medical Oncology, The University of Texas MD Anderson Cancer Center, Houston, TX, USA; ^3^ Department of Translational Molecular Pathology, The University of Texas MD Anderson Cancer Center, Houston, TX, USA; ^4^ Department of Biostatistics, The University of Texas MD Anderson Cancer Center, Houston, TX, USA; ^5^ Melanoma Medical Oncology, The University of Texas MD Anderson Cancer Center, Houston, TX, USA; ^6^ Genomic Medicine, The University of Texas MD Anderson Cancer Center, Houston, TX, USA; ^7^ Cancer Biology, The University of Texas MD Anderson Cancer Center, Houston, TX, USA

**Keywords:** ISG15, USP18, PTEN, protein stability, and lung cancer

## Abstract

The ubiquitin-like modifier interferon-stimulated gene 15 (ISG15) is implicated in both oncogenic and tumor suppressive programs. Yet, few ISGylation substrates are known and functionally validated in cancer biology. We previously found specific oncoproteins were substrates of ISGylation and were stabilized by the ISG15-specific deubiquitinase (DUB) ubiquitin specific peptidase 18 (USP18). Using reverse-phase protein arrays (RPPAs), this study reports that engineered loss of the DUB USP18 destabilized the tumor suppressor protein phosphatase and tensin homologue (PTEN) in both murine and human lung cancer cell lines. In contrast, engineered gain of USP18 expression in these same lung cancer cell lines stabilized PTEN protein. Using the protein synthesis inhibitor cycloheximide (CHX), USP18 knockdown was shown to destabilize PTEN whereas USP18 overexpression stabilized PTEN protein. Interestingly, repression of USP18 decreased cytoplasmic PTEN relative to nuclear PTEN protein levels. We sought to identify mechanisms engaged in this PTEN protein destabilization using immunoprecipitation assays and found ISG15 directly conjugated with PTEN. To confirm translational relevance of this work, USP18 and PTEN immunohistochemical expression were compared in comprehensive lung cancer arrays. There was a significant (*P* < 0.0001) positive correlation and association between PTEN and USP18 protein expression profiles in human lung cancers. Taken together, this study identified PTEN as a previously unrecognized substrate of the ISGylation post-translational modification pathway. The deconjugase USP18 serves as a novel regulator of PTEN stability. This indicates inhibition of ISGylation is therapeutically relevant in cancers.

## INTRODUCTION

Lung cancer is the most common cause of cancer death for men and women [1]. Despite current treatments, lung cancer often becomes resistant to therapies [2], driving investigators to uncover alternative ways to target oncoproteins required for tumorigenesis. Recently, pathways involved in protein homeostasis have proven to play critical roles in cancer biology and therapy [3]. These pathways include ubiquitin and ubiquitin-like modifiers (ULMs) that have ability to modify protein function through protein degradation and other processes [4]. Post-translational modifications (PTMs) that affect protein stability are attractive processes to target the functions of oncoproteins or tumor suppressors that exert rate-limiting roles in carcinogenesis [3, 4].

The phosphatase and tensin homologue (PTEN) protein is a major tumor suppressor commonly lost in multiple cancer-types [5]. PTEN functions as a phosphatase for tyrosine and serine/threonine residues on phosphatidylinositol (3,4,5)-triphosphate (PIP3). This can inhibit downstream signaling to protein kinase B (AKT) and affect rapamycin (mTOR)-dependent pathways [5]. Dephosphorylation of PIP3 results in repression of cell growth, reduction of cell cycle progression, and increased apoptosis in cancer cells [5]. In lung cancer, somatic mutations in *PTEN* are found in only 4-8% of cases [6]. Even though *PTEN* mutations are rare in lung cancer, PTEN protein is often lost in these tumors [7]. Interestingly, there is little correlation between *PTEN* mRNA and PTEN protein expression in studied cancers, implying that aberrant post-transcriptional or post-translational regulators of PTEN are engaged for its repression in tumorigenesis [8]. Prior work revealed that PTEN is post-translationally regulated by modifications such as phosphorylation, acetylation, oxidation, s-nitrosylation, and ubiquitination [8]. Recent work found the deubiquitinase (DUB) ubiquitin specific peptidase 7 (USP7) controls subcellular localization of PTEN and its proteasomal degradation [9]. In contrast, the ubiquitin specific peptidase 13 (USP13) was the only DUB yet uncovered to stabilize PTEN [10]. This implicated that stability and activity of PTEN and other proteins involved in oncogenesis are altered by specific DUBs. Understanding these processes will not only provide insights into PTEN biology, but also advance our understanding of how this critical growth-regulatory protein can affect carcinogenesis.

The interferon-stimulated gene 15 (ISG15) protein was the first ULM identified and was initially identified as part of ubiquitin-stimulated immune response [11]. Similar to ubiquitin, ISG15 conjugation is activated by a cascade consisting of an E1-activating protein (UBE1L), an E2-conjugating protein (UBCH8), and an E3-ligase (most commonly HERC5), which facilitates ISG15 transfer to protein substrates [12–14]. The major deubiquitinating enzyme ubiquitin specific peptidase 18 (USP18) can remove ISG15 from its target proteins, reversing effects of ISGylation [15]. Studies showed that ISGylation results in destabilization of specific protein substrates [16–20]. The precise consequences of ISGylation are being elucidated, but there is growing evidence that this pathway has specialized functions [18]. We previously showed that engineered loss of USP18 results in the destabilization of specific oncoproteins [19–20 and LM Mustachio personal communication]. Recent work also uncovered a potential tumor suppressive role for USP18 in *FVB-Usp18* knockout mice [21]. Thus, identifying whether ISGylation was tumor promoting or suppressive prompted us to conduct studies to discern critical growth-regulatory pathways engaged by ISGylation.

The experiments reported here indicate the PTEN tumor suppressor is a previously unrecognized target of ISGylation. Reverse-phase protein arrays (RPPAs) of lung cancer cell lines engineered with repressed USP18 expression uncovered PTEN as a potential target of the ISGylation pathway. Immunoblotting of PTEN levels in murine and human lung cancer cell lines with engineered knockdown versus overexpression of USP18 (as compared to controls) established USP18 as a regulator of PTEN protein levels and stability. Immunoprecipitation (IP) assays confirmed that ISG15 directly complexed with PTEN protein and this conjugation was attenuated by engineered overexpression of USP18. The translational relevance of this work was confirmed in human lung cancer arrays that revealed USP18 and PTEN immunostaining were positively correlated. These findings not only contribute to the understanding of the functional consequences of ISGylation, but also advance our knowledge of how PTEN is post-translationally regulated in tumorigenesis.

## RESULTS

### USP18 and ISG15 regulate PTEN protein

Putative ISGylation substrates have been uncovered, however few were confirmed as direct protein targets [22]. We previously identified specific ISGylation substrates involved in oncogenesis by manipulating expression of the ISG15-specific protease USP18 [19,20]. Since the functions of ISGylation are under active study, we aimed to identify new target proteins of the pathway. Both ED1 murine and HOP62 human lung cancer cell lines engineered with stable knockdown of USP18 were used to monitor expression of 304 growth-regulatory proteins that contribute to tumorigenesis in an RPPA (Supplementary Table S1 and Supplementary Figure S1). It was hypothesized that differential expression of these species accompanied loss of USP18 expression and accompanied the observed reduction of lung cancer cell growth (Supplementary Figure S2).

**Figure 1:**
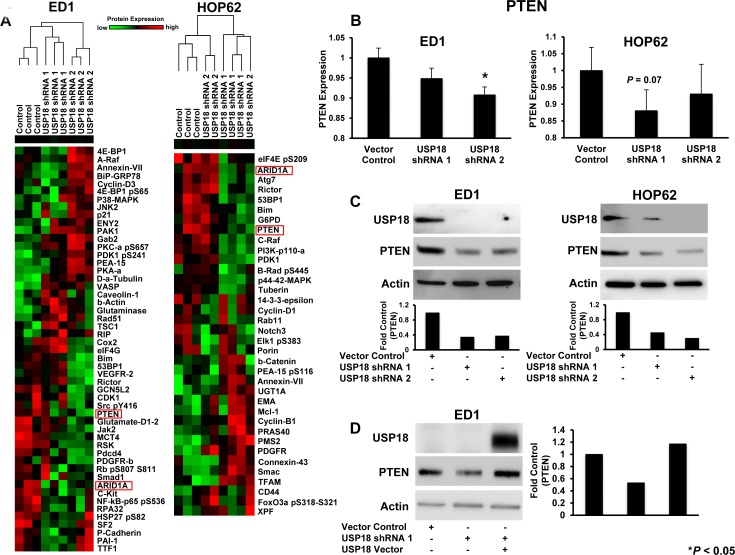
RPPA-based protein profiling revealed USP18 regulated PTEN levels **A.** Independent RPPAs are displayed of ED1 and HOP62 lung cancer cell lines stably transfected with a control vector or one of two individual shRNAs against USP18. Signal intensities were normalized and transformed to linear values. RPPA highlighted species showing substantial differences in expression profiles between vector control and USP18 shRNA transfected lung cancer cells are represented by heat-maps for both ED1 and HOP62 lung cancer arrays. Red indicated high and green indicated low protein expression. Cell line clusters are shown as dendrograms. PTEN (red box) was highlighted as one of the proteins showing similar trends in both arrays. **B.** PTEN expression in the displayed ED1 and HOP62 transfected lung cancer cell lines was quantified using normalized linear values. Cell lines stably transfected with shRNAs against USP18 were compared relative to vector control transfectants. **C.** Representative immunoblots independently probed with USP18 and PTEN recognizing antibodies confirmed that engineered repression of USP18 decreased PTEN protein levels in ED1 and HOP62 lung cancer cells. PTEN expression was normalized to actin expression and compared between vector control and USP18 shRNA-transfected lung cancer cell lines. **D.** Decreased PTEN protein levels caused by loss of USP18 expression were rescued by transiently overexpressing GFP-tagged USP18 in ED1 lung cancer cells. Immunoblots were individually probed with GFP and PTEN recognizing antibodies. PTEN was normalized to actin expression and compared between vector control and USP18 shRNA- as well as USP18 shRNA/USP18 vector-transfected lung cancer cell lines.

Diverse proteins on this array were affected by USP18 knockdown. Only a small number of these proteins showed substantial differences in expression profiles between USP18 knockdown and vector controls (Figure 1A). To identify new potential targets from amongst these highlighted proteins, murine and human lung cancer cell lines were individually analyzed to determine consistent expression trends between two independent shRNAs that each conferred USP18 repression. Expression of proteins that showed consistent changes were compared between murine and human lung cancer cell lines using RPPAs to determine whether similar trends existed between these lines. The tumor suppressors PTEN [5] and AT-rich interactive domain-containing protein 1A (ARID1A) [23] were the only proteins that showed consistent expression changes between these shRNA-transfected groups using these arrays. Both PTEN and ARID1A expression decreased with engineered USP18 loss (Figure 1B and Supplementary Figure S3).

*ARID1A* exhibits up to a 50% mutation frequency in diverse cancers [24]. Studies examining mRNA and protein levels indicated that decreased *ARID1A* mRNA correlates with reduced ARID1A protein levels in the majority of tumors analyzed with *ARID1A* mutations [25, 26]. In contrast, *PTEN* mRNA and protein are altered despite genetic alterations in the *PTEN* gene [27]. This observation is supported by evidence from The Cancer Genome Atlas (TCGA), which revealed while *PTEN* was mutated or altered in only ~2% of lung adenocarcinoma cases (Supplementary Figure S4A), *PTEN* mRNA levels were significantly lower in 517 cases of lung adenocarcinomas as compared to normal lung tissues (Supplementary Figure S4B). Since prior work indicated there is rarely a direct correlation between *PTEN* mRNA and PTEN protein levels [8] and because ISGylation was a PTM not yet shown to regulate PTEN [8], attention next focused on PTEN and its regulation by ISGylation in lung cancer.

**Figure 2:**
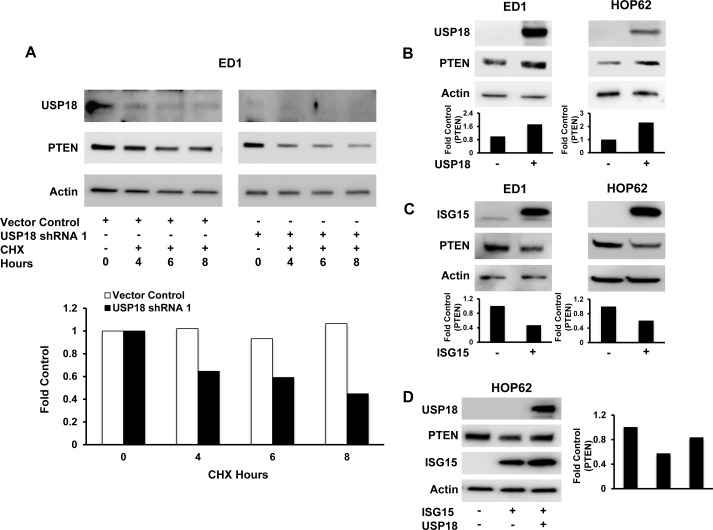
Modifying USP18 and ISG15 protein levels altered PTEN protein stability **A.** Loss of USP18 expression decreased PTEN protein stability. ED1 murine lung cancer cells stably transfected with a vector control or with one of two individual shRNAs against USP18 were subjected to an 8 hour CHX treatment. PTEN expression was quantified relative to respective actin expression at indicated time points and normalized to the 0 hour (before CHX treatment) time point. **B.** Gain of USP18 expression increased PTEN levels. ED1 and HOP62 lung cancer cells were independently transfected with a USP18 expression plasmid or a control vector. PTEN expression was normalized to actin expression and compared between vector control and USP18 vector-transfected lung cancer cell lines. **C.** Engineered gain of ISG15 expression decreased PTEN protein levels. ED1 and HOP62 lung cancer cells were independently transfected with ISG15 or a vector control. PTEN was normalized to actin expression and compared between vector control and ISG15 vector-transfected lung cancer cell lines. **D.** Decreased PTEN levels conferred by overexpressed ISG15 were rescued by transiently overexpressing GFP-tagged USP18 in HOP62 lung cancer cells. Immunoblots were independently probed with GFP and PTEN recognizing antibodies. PTEN protein levels were normalized to actin expression and compared between vector control and ISG15 vector as well as ISG15 vector/USP18 vector-transfected lung cancer cell lines.

To confirm and extend these RPPA results, immunoblot experiments were performed. These studies revealed repression of USP18 by independent siRNAs (Supplementary Figure S5A) and shRNAs (Figure 1C and Supplementary Figure S5B) downregulated PTEN levels in both murine and human lung cancer cell lines. Quantification of PTEN protein levels after USP18 repression in ED1 and HOP62 lung cancer cell lines revealed a reduction of PTEN protein by at least 50% as compared to vector control cells. As expected, this downregulation of PTEN was at the protein and not mRNA level (Supplementary Figure S5C). Restoration of USP18 levels in USP18-repressed cells rescued PTEN expression as compared to vector transfected controls (Figure 1D). Since USP18 is known to regulate stability of ISG15 substrate proteins [16, 28], we analyzed the effect of USP18 loss on PTEN stability over an 8 hour course of cycloheximide (CHX) treatment. When USP18 expression was repressed in the ED1 murine lung cancer cell line, only ~44% of PTEN protein was stabilized after 8 hours of CHX treatment as compared to controls (Figure 2A). In contrast to USP18 repression studies, when USP18 was overexpressed in HOP62 human lung cancer cell lines, PTEN levels increased by over 70% compared to vector control transfected cells (Figure 2B). When overexpressed, USP18 stabilized PTEN protein levels after 6 hours of CHX treatment, as compared to vector control transfectants that showed a reduction in PTEN levels (Supplementary Figure S6). Since USP18 removes ISG15 from its target proteins, it was hypothesized that gain of ISG15 expression had a similar effect on PTEN as did USP18 loss. This possibility was supported by previous work indicating that overexpression of ISG15 decreased expression of its target proteins [29]. Indeed, engineered overexpression of ISG15 decreased PTEN levels in both murine and human lung cancer cell lines by at least ~40-50% as compared to vector control transfected cells (Figure 2C). When USP18 was simultaneously overexpressed with ISG15, PTEN levels were rescued and were similar to levels detected in control cells (Figure 2D).

**Figure 3:**
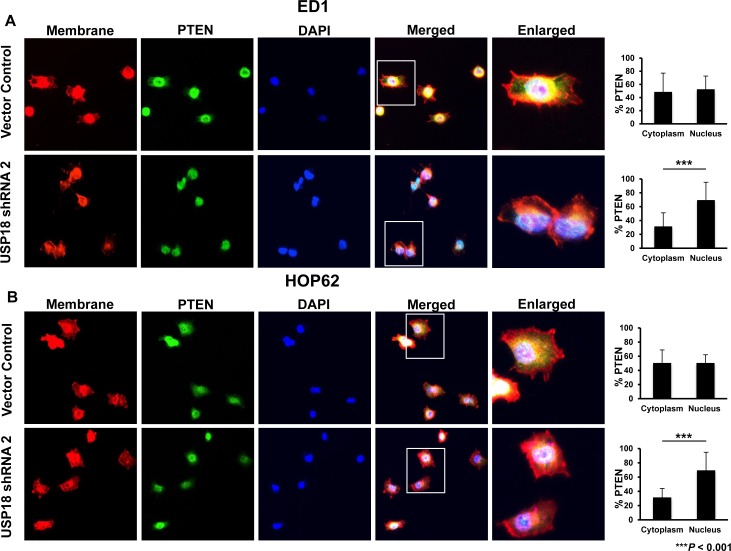
Loss of USP18 expression decreased cytoplasmic PTEN protein **A.** ED1 and **B.** HOP62 lung cancer cell lines stably transfected with a vector control or shRNA against USP18 were fixed and stained for PTEN, sodium potassium ATPase, and DAPI. Cells were imaged by confocal microscopy and the magnification was 63X. Percent immunostaining of PTEN in the cytoplasm and nucleus was quantified and compared. Data are representative of 20 cells per group for each analyzed experiment.

These data indicated that loss of USP18 destabilized PTEN protein. USP18 is predominantly a cytoplasmic protein [30]. Since other DUBs and PTMs are known to regulate cytosolic stability of PTEN protein [10, 31], it was hypothesized that loss of USP18 would likely affect cytoplasmic PTEN protein levels. Fluorescent microscopy revealed control cells contained similar proportions of PTEN in the nucleus and cytoplasm in murine (Figure 3A) and human (Figure 3B) lung cancer cell lines. In marked contrast, murine and human lung cancer cell lines having repressed USP18 expression profiles significantly (*P* < 0.001) decreased cytosolic relative to nuclear PTEN protein levels (Figure 3A-3B). While PTEN protein levels in the cytosol decreased in lung cancer cell lines with repressed USP18 expression (compared to vector control), PTEN levels in the nucleus remained similar between vector control and USP18 shRNA-expressing lung cancer cell lines (Supplementary Figure S7A and B). This finding established that loss of USP18 expression reduced cellular PTEN levels and promoted destabilization of cytosolic PTEN [10, 31]. This observation supports prior work that revealed USP18 regulated destabilization of ISG15 complexed proteins in the cytosol [32, 33]. We next uncovered mechanisms responsible for these interactions by analyzing whether ISG15 directly conjugated to PTEN.

### PTEN ISGylation

**Figure 4:**
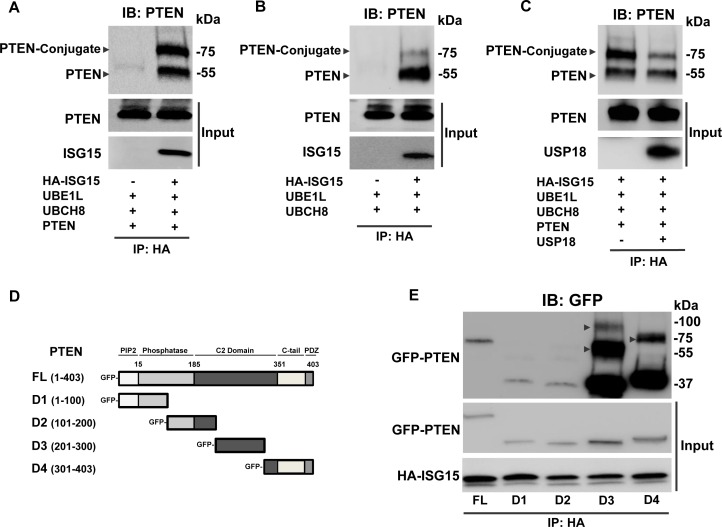
PTEN undergoes ISGylation ISG15 conjugates to PTEN in lung cancer cell lines. Immunoprecipitation (IP) with an anti-HA antibody followed by immunoblot (IB) with an anti-PTEN antibody revealed ISG15-PTEN complex formation between HA-tagged ISG15 and **A.** exogenous and **B.** endogenous PTEN at ~75 kDa in HOP62 lung cancer cells. **C.** The ISG15-PTEN conjugation observed by IP was attenuated with introduction of USP18 in HOP62 lung cancer cells. **D.** Deletions of PTEN at indicated domains were subjected to **E.** IP by HA-tagged ISG15 using an anti-HA antibody followed by IB with an anti-GFP antibody revealed differential conjugation between PTEN deletions in ED1 lung cancer cells. Arrows displayed ISG15-PTEN conjugation bands.

USP18 is the major ISG15-specific protease that can deconjugate ISG15 from substrate proteins [15]. Since PTEN expression is altered by regulated USP18 expression, PTEN was hypothesized to be a substrate of ISGylation. IP assays of human lung cancer cells transfected with HA-tagged ISG15 revealed that both exogenous (Figure 4A and Supplementary Figure S8) and endogenous (Figure 4B) PTEN proteins are mono-ISGylated resulting in the expected molecular weight ~75 kDa species. When gain of USP18 expression was achieved, expression of the putative ISG15-PTEN conjugated species decreased (Figure 4C).

To determine PTEN domains that were ISG15 modified, deletion constructs of PTEN were engineered (Figure 4D). IP experiments revealed pull-down of all PTEN deletions (Figure 4E). This finding was supported by prior work that showed ISG15 modifies multiple regions of a protein substrate and that ISG15-conjugated PTEN was a mixture of ISG15 species non-specifically bound to different amino acid residues of PTEN [34]. ISG15 can mono-ISGylate its protein substrate at different residues simultaneously, accounting for shifts in ISG15 modified conjugation [33, 34]. Interestingly, PTEN deletions D3 (the C2 region of PTEN) and D4 (the C-tail region of PTEN) exhibited prominent conjugation to ISG15 at molecular weights that together indicated that ISG15 mainly binds to the C-terminus of PTEN. These conjugation bands were more evident than observed for full-length PTEN protein. Other PTEN deletions were only detected after longer exposures. The native PTEN protein confirmation might hinder ISG15 conjugation to some residues, allowing ISG15 to complex more efficiently when some PTEN domains are deleted [31]. These data indicated that ISG15 directly associates with PTEN.

### PTEN and USP18 associations in human lung cancers

**Figure 5:**
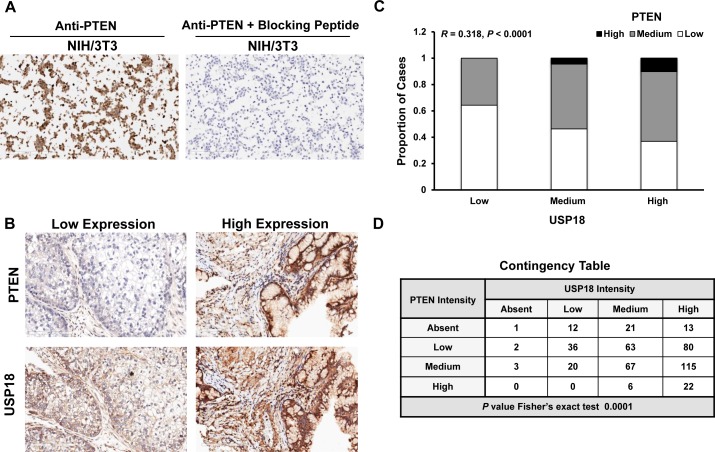
PTEN and USP18 expression profiles are associated in human lung cancers **A.** Blocking peptide antagonized PTEN immunostaining in the NIH/3T3 (PTEN positive) cell line. **B.** Representative staining for human lung cancers with low PTEN and USP18 or high PTEN and USP18 expression profiles. All magnifications were 20X. **C.** PTEN and USP18 immunostaining are positively correlated in human lung cancer (*n* = 461) and **D.** This association was confirmed using a contingency table by grouping negative, low, medium, or high PTEN and USP18 immunostaining profiles in lung cancer cases.

An association was established between PTEN and USP18 proteins *in vitro*. It was next determined if these findings translated into human lung cancer cases. A human lung cancer microarray was immunostained for PTEN and USP18. Specificity of the PTEN antibody was confirmed using a blocking peptide (Figure 5A and Supplementary Figure S9). Immunostaining profiles of representative lung cancer cases are displayed (Figure 5B). Based on these data, it was proposed that cases with high levels of USP18 also had high levels of PTEN. Of 507 lung cancer cases evaluated in the lung cancer array, analyses were conducted in 461 cases that were adequately immunostained for both PTEN and USP18 expression profiles. These cases showed a statistically significant (*P* < 0.0001) correlation between PTEN and USP18 expression in human lung cancers (Figure 5C). PTEN and USP18 expressing lung cancer cases were examined for independent groups based on absent, low, medium, or high PTEN and USP18 immunostaining profiles. Based on the displayed contingency table, lung cancer cases with high PTEN expression exhibited high USP18 levels (Figure 5D). Together, these data confirmed that USP18 affected PTEN expression in human lung cancers.

## DISCUSSION

PTMs control key signals during carcinogenesis by determining expression profiles of growth-regulatory oncogenic and tumor suppressive proteins [34]. The PTM ISG15 has distinct functions and is regulated by the DUB USP18 [15,18,35]. Both ISG15 [36] and USP18 [19] are deregulated in different cancers, consistent with an important functional role for this DUB in homeostasis of growth-regulatory proteins. This view is supported by our prior work that established ISGylation affects stability of key growth-regulatory proteins in acute promyelocytic leukemia (APL) and lung cancer [16, 17, 19, 20]. Improved understanding of the functional roles played by ISGylation should advance our knowledge of protein destabilization pathways that control oncogenesis by precisely regulating intracellular oncoprotein or tumor suppressor proteins.

In the search for novel targets of ISGylation in lung cancer, RPPAs uncovered PTEN as a previously unrecognized candidate ISG15 substrate. This extended prior work by showing USP18 regulates protein levels of the PTEN tumor suppressor. The frequent loss of PTEN at the mRNA and protein levels as compared to its infrequent mutations (when coupled with the lack of correlation between *PTEN* mRNA and PTEN protein) indicated the importance of PTMs in regulating PTEN in oncogenesis [8]. Given the importance of regulating PTEN levels, it is not surprising a DUB other than USP7 and USP13 [9,10] affects the stability of this tumor suppressor. The key observations presented here are summarized in Supplementary Figure S10.

Engineered loss versus gain of USP18 expression respectively decreased and increased PTEN protein levels and stability in murine and human lung cancers. Yet, manipulation of USP18 levels did not alter *Pten* mRNA levels, indicating that USP18 regulated PTEN post-transcriptionally. Since prior work established that ISGylation affects stability of complexed proteins [16, 17], IP assays were performed and found that ISG15 can directly conjugate with PTEN. The presented data indicated that several PTEN sites were potentially ISGylated. Similar findings were previously reported with other proteins that are ISG15-modified at both N- and C-terminal domains [33]. Given that USP18 and PTEN profiles are significantly (*P* < 0.0001) associated in human lung cancers, it is not surprising that the ISGylation can affect PTEN expression in malignant tissues.

ISG15 is known to destabilize substrate proteins through the 20S proteasome [34]. Similar to USP13 [10], USP18 is primarily located in the cytoplasm, consistent with its regulation of cytoplasmic PTEN [30]. Data displayed here indicate that loss of USP18 induced destabilization of PTEN protein in the cytoplasm. This was observed with other PTMs like ubiquitination, which complex with and destabilize cytosolic PTEN protein [31]. Since PTMs can affect PTEN localization [9] and ISG15 is able to control localization of its substrate proteins [35, 36], it is possible that in addition to destabilizing PTEN, ISG15 imports PTEN to the nucleus. Total PTEN levels decreased with USP18 loss and PTEN levels in the nucleus (between vector control and USP18 shRNA-transfected lung cancer cells) did not appreciably change. It was therefore not surprising that repression of USP18 augmented PTEN cytoplasmic destabilization. Nuclear PTEN plays a critical role in chromosome stability, DNA repair, cell cycle arrest, and cellular stability [37]. In neoplastic tissues, cytoplasmic PTEN predominates, but nuclear PTEN could exert tumor-suppressive activity [37]. Considering there is more abundant PTEN protein expressed in the nucleus than in the cytosol after engineered loss of USP18, it is hypothesized the tumor-suppressing ability of PTEN is affected [37]. Future work will identify the precise functional consequences of PTEN destabilization conferred by engineered loss of USP18 expression.

Ubiquitin-like pathways and specific DUBs are reported to affect both oncogenes and tumor suppressors [38, 39]. In some studies, ISG15 functioned as a tumor suppressor as its expression was increased by antineoplastic agents like all-*trans*-retinoic [20, 40]. In other studies, ISG15 was identified as an oncoprotein because it was often overexpressed in specific cancer-types and negatively regulated expression of tumor suppressors such as p53 [33, 40]. We previously reported that USP18 expression was increased in malignant versus normal lung tissues [19]. Likewise, engineered loss of USP18 expression decreased lung cancer cell growth and increased apoptosis in these cancer cells [19 and LM Mustachio personal communication]. Our prior work found that USP18 affected stability of cyclin D1 and KRAS, proteins that are overexpressed or constitutively activated in lung cancer cases [19 and LM Mustachio personal communication]. In contrast, engineered loss of *Usp18* led to development of leiomyosarcomas, but this is a strain specific effect [21]. Thus, whether USP18 exerts an oncogenic or tumor suppressive effect depends on the tissue and cell contexts. Yet, current evidence indicates that oncogenic signals of USP18 appear most frequent [19–21 and LM Mustachio personal communication]. To elucidate which function predominates will require the development and characterization of USP18 inhibitors.

Different cellular and tumor contexts determine whether a DUB has a net oncogenic or tumor suppressive effect [38]. For example, USP7 studies found DUBs can exert distinct functional effects based on the substrate protein abundance and the cell type and physiological state [39]. In different cancers, consequences of USP18 repression on PTEN or other ISG15ylated proteins might differentially affect downstream signaling pathway [39]. There are likely growth-regulatory proteins upstream or downstream of PTEN that exert oncogenic functions and are affected by USP18 loss. An example of this is found in sarcoma where dual repression of PTEN and p53, another ISGylation substrate [33], can promote cancer progression [41]. Thus, classifying USP18 as an oncogenic or tumor suppressive species is likely cancer specific. Whether USP18 should be targeted to confer antineoplastic effects is a priority of future work.

This study elucidated PTEN as a new substrate of ISGylation. Additional studies are needed to delineate the precise functional consequences of this ISGylation, uncover other ISGylated substrates, and learn in which cancers USP18 exerts a net oncogenic or tumor suppressive effect. As these studies unfold, it is important to keep in mind that engineered loss of USP18 does reduce lung cancer formation in mouse models (19 and L.M. Mustachio personal communication). This is why identifying an inhibitor of USP18 is an important next step in the investigations of this DUB. Likewise, it will be necessary to discover the antineoplastic effects of an inhibitor of USP18. Such an inhibitor could act as a single agent or in combination with another chemotherapeutic agent in lung and potentially other cancers.

## MATERIALS AND METHODS

### Cell culture

Murine (ED1 and LKR13) and human (HOP62 and H522) lung cancer cell lines were cultured in RPMI 1640 medium (Invitrogen) supplemented with 10% fetal bovine serum (FBS) (Thermo Scientific). 293T cells were cultured in DMEM medium (Invitrogen) supplemented with 10% FBS. Cells were cultured at 37°C in a humidified incubator with 5% CO_2_. Cell lines were obtained from American Type Culture Collection (ATCC) except for ED1 [42] and LKR13 cells [43].

### Plasmids and SiRNAs

The plasmids pCMV-HA-ISG15, pSG5-UBE1L, pCMV2-UBCH8, and pcDNA4-USP18 were described before [16, 19]. Full-length PTEN cloned into a MYC-tagged expression vector [10] was obtained from Dr. Li Ma (MD Anderson Cancer Center). The pcDNA3-GFP-PTEN and the pCMV-GFP-USP18 plasmids were purchased (Addgene and GeneCopoeia). Deletions in PTEN were constructed (GENEWIZ). DNA sequence analysis confirmed the desired species were engineered. Respective vector controls were purchased. RISC-free control siRNA and two siRNAs independently targeting USP18 were purchased (GE Dharmacon). These sequences were: murine USP18 siRNA 1 (5′-CGTTGTTTGTCCAGCACGA-3′) and murine USP18 siRNA 2 (5′-AGGAACTCGAGGACGGAAA-3′). Plasmids and siRNAs were transfected into cells using Lipofectamine 2000 reagent (Invitrogen) and Opti-MEM medium (Gibco Thermo Scientific).

### Immunoprecipitation and immunoblot analyses

Cells were washed with phosphate buffered saline (PBS) (Corning) and lysed with ice-cold Pierce RIPA Lysis and Extraction Buffer (Thermo Scientific) supplemented with Halt Protease and Phosphatase Inhibitor Cocktail (Thermo Scientific). IP reactions were completed with the Pierce HA-Tag IP/Co-IP Kit (Thermo Scientific). Proteins were resolved on SDS-PAGE before transfer to nitrocellulose membranes (Whatman). Membranes were blocked in 5% nonfat milk dissolved in Tris-buffered saline with 0.1% Tween 20 for at least 1 hour before overnight incubation at 4°C with primary antibody diluted in 5% nonfat milk or 5% bovine serum albumin. This was followed by 40 minute incubation with secondary antibody diluted in 5% nonfat milk at room temperature. Antibody binding was visualized by Luminata Forte (EMD Millipore) and quantified by ImageJ software (version 1.45s, imagej.nih.gov/ij). Antibodies used for immunoblot analyses were: anti-USP18 (Cell Signaling #4813), anti-ISG15 (Cell Signaling #2743), anti-ß-Actin (Cell Signaling #3700), anti-GFP (Santa Cruz Biotechnology #sc-8334), and anti-PTEN (Cell Signaling #9552). Two different antibodies were derived to recognize USP18 [19, 20]. Secondary anti-mouse and anti-rabbit antibodies were purchased (Amersham Biosciences). Immunoblots were stripped using Restore PLUS Western Blot Stripping Buffer (Thermo Scientific). To analyze for stability of PTEN, cells were treated with 60 μg/ml of CHX (Sigma-Aldrich) for 6 to 8 hours.

### Quantitative real-time PCR assays

For qRT-PCR assays, primers were: murine *Pten* forward primer (5′-AACTTGCAATCCTCAGTTTG-3′) and reverse primer (5′-CTACTTTGATATCACCACACAC-3′); murine *Gapdh* forward primer (5′-AGGTCGGTGTGAACGGATTTG-3′) and reverse primer (5′-TGTAGACCATGTAGTTGAGGTCA-3′). Assays were performed using previously optimized methods [19, 20].

### Stable cell lines with repressed USP18 expression

Lentiviral pCMV-dR8.2 dvpr and pMD2.G plasmids were purchased (Addgene). Five candidate TRC pLKO.1 lentiviral shRNAs repressing USP18 were purchased (GE Dharmacon). The different shRNA lentiviral particles used for individual USP18 knockdown experiments in murine and human cell lines were generated in 293T cells, as before [19]. Repression of USP18 was confirmed by immunoblot analyses. Two shRNAs with the greatest knockdown efficiency were individually used in the displayed experiments.

### Immunofluorescence

ED1 and HOP62 lung cancer cells with stable knockdown of USP18 were cultured on coverslips (Fisherbrand). After 24 hours, cells were washed (three times for five minutes each with PBS) and fixed with 4% paraformaldehyde (PFA) (Electron Microscopy Sciences) for 20 minutes at room temperature. Cells were washed again and permeabilized with 0.1% TritonX-100 (Fisherbrand) for 5 minutes at room temperature before blocking with 3% bovine serum albumin (PBS-BSA) for 1 hour at room temperature. Cells were incubated with a murine monoclonal antibody that recognized PTEN (Thermo Scientific #32-5800) or a murine monoclonal antibody isotype control (Biolegend #401602) diluted at 1:10 in 3% PBS-BSA and rabbit monoclonal anti-Sodium Potassium ATPase (Alexa Fluor 647) antibody (Abcam #198367) diluted at 1:100 in 3% PBS-BSA overnight at 4°C. The following day, cells were washed and incubated with a FITC-conjugated goat anti-mouse IgG secondary antibody (Thermo Scientific #F2761) diluted at 1:1000 in 3% PBS-BSA for 1 hour in the dark at room temperature. Cells were washed before mounting on coverslips (Fisherbrand) with Prolong Gold Anti-Fade Reagent with DAPI (Thermo Scientific). Fluorescence was examined using a confocal microscope (Leica Microsystems) and quantitated by using ImageJ (Fiji) software (version 2.0.0-rc-43/1.50e).

### Reverse-phase protein array

RPPA analyses were performed in cell lysates from ED1 and HOP62 lung cancer cells stably transfected with vector control or with one of two individual USP18 shRNAs and subsequently analyzed in the RPPA core facility at MD Anderson Cancer Center. Samples were serially diluted and probed with 304 antibodies and arrayed on nitrocellulose-coated slides. Relative protein levels were normalized for protein loading and determined by interpolation of each dilution curve from the standard curve [44]. Normalized data points were transformed to a linear value used for analysis.

### The cancer genome atlas

Expression and mutation data for lung adenocarcinomas and expression data for normal lung tissues were obtained from public repositories of TCGA. To compare expression levels, transcripts per million units were used, which was previously found as optimal for comparing expression data from RNA sequencing [45].

### Immunohistochemistry

A lung cancer microarray was developed at MD Anderson Cancer Center. Primary non-small cell lung cancer cases (*n* = 507) from surgically resected lung tumors were used in this array. Immunostaining was performed on formalin-fixed paraffin-embedded sections using a Leica BOND-MAX^TM^ automated stainer and Leica Bond Polymer Refine Detection reagents (Leica Microsystems) to detect USP18 expression using a described antibody [19, 20] and PTEN expression using a purchased antibody (Cell Signaling #9188). Specificity of the USP18 antibody was shown with a blocking peptide [19]. Specificity of the PTEN antibody was determined with a PTEN immunostaining control (Cell Signaling #8106) and a blocking peptide (Cell Signaling #1250). Slides were digitally scanned using an Aperio AT2 scanner (Leica Biosystems). USP18 and PTEN immunostaining profiles were scored with absent (0), low (1-100), medium (101-200), or high (201-300) expression levels, as before (21). Statistical software GraphPad Prism and R were used for analyses.

### Study approval

The Institutional Review Board reviewed and approved studies conducted with human lung cancer microarrays.

### Statistics

Two-tailed Student's *t* test compared differences between two studied groups. Spearman rank correlation measured the strength of association between two variables. Fisher's exact test evaluated the association between two categorical variables. Results of independent experiments were pooled to assess statistical significance. Statistical significance was defined as *P* < 0.05, unless otherwise noted.

## SUPPLEMENTARY MATERIAL




