# Supplementary material for: The ISG15-specific protease USP18 regulates stability of PTEN
Source: Oncotarget. 2016 Dec 12;8(1):3–14. doi: 10.18632/oncotarget.13914 (PMC5352120; doi:10.18632/oncotarget.13914)
Supplement: Supplementary file 1 [file oncotarget-08-3-s001.pdf]

The ISG15-specific protease USP18 regulates stability of PTEN

Supplementary Material

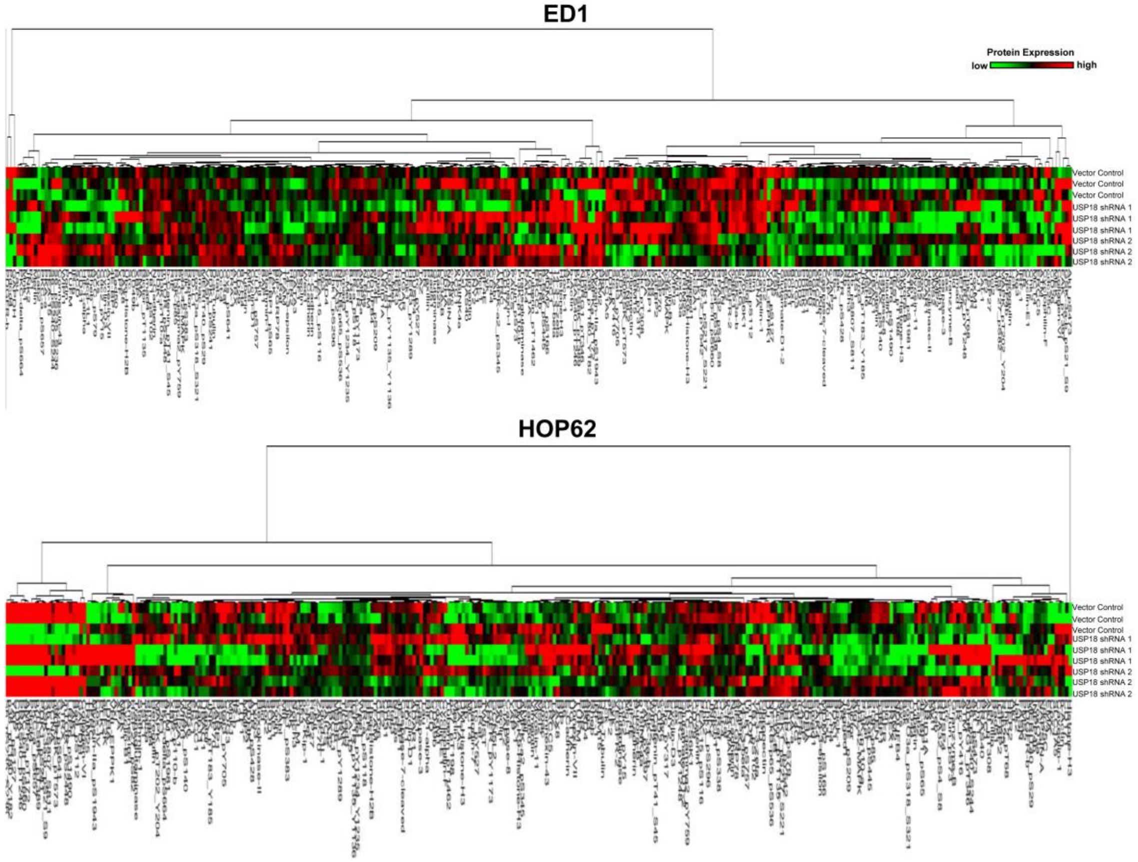

Supplementary Figure S1: RPPA heat-maps for USP18-repressed murine and human lung cancer cell lines. Expression profiles are shown for the 304 growth-regulatory proteins in ED1 and HOP62 lung cancer cell lines that were stably transfected with a control vector or individual shRNAs that target USP18 for repression. Signal intensities were normalized and transformed to linear values. Red indicates high and green displays low protein expression. Protein clusters are shown as dendrograms.

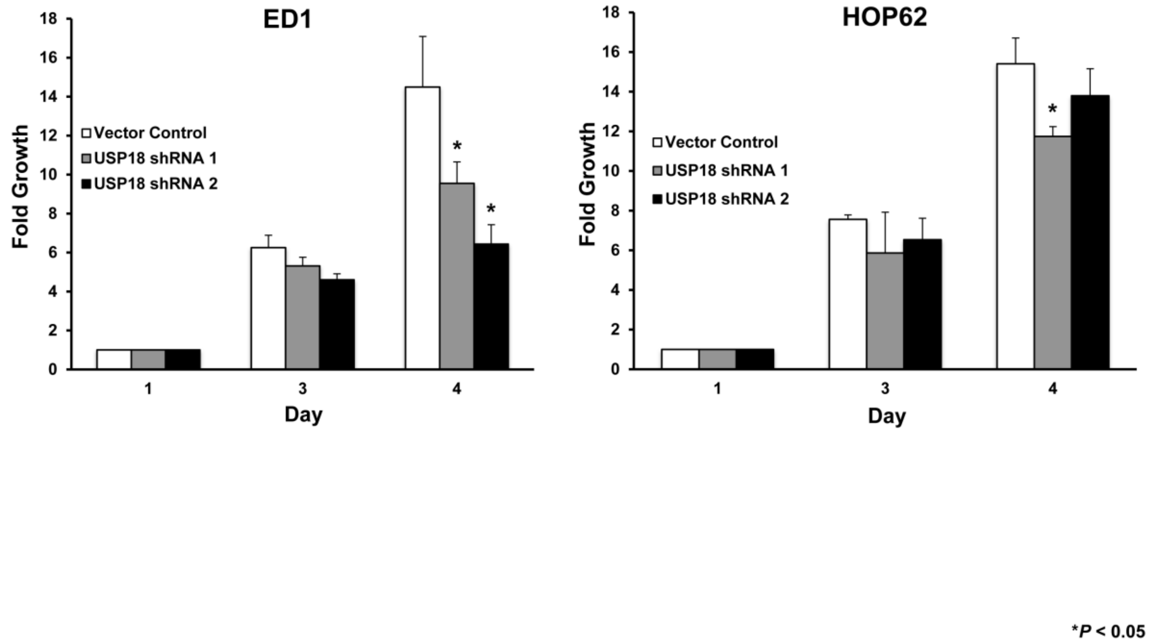

Supplementary Figure S2: Loss of USP18 reduced cell growth of murine and human lung cancer cells. Cell growth was monitored over a 4 day study. All cell lines were normalized to their respective proliferation on day 1.

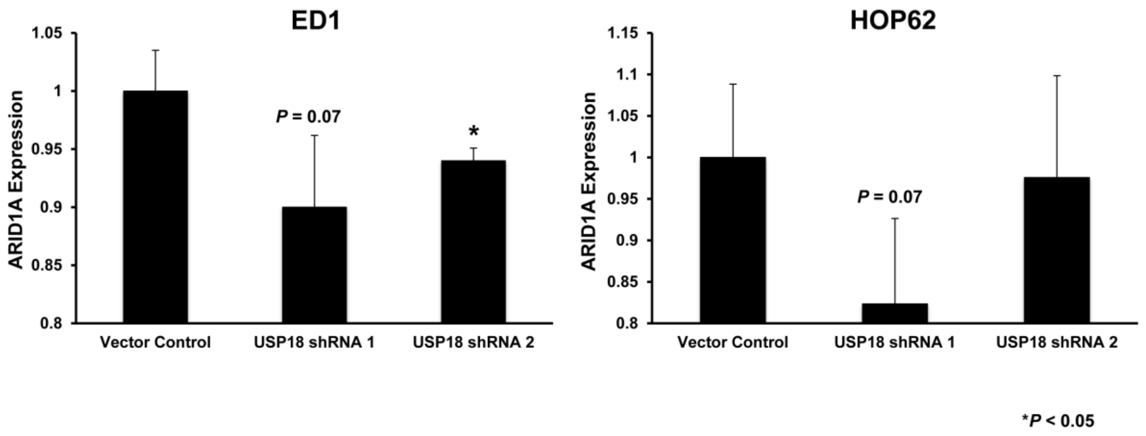

Supplementary Figure S3: Repression of USP18 decreased ARID1A protein levels. ARID1A expression profiles determined by RPPAs in ED1 and HOP62 lung cancer cell lines were quantified using normalized linear values. These lung cancer cell lines stably transfected with individual shRNAs that reduce USP18 expression were compared relative to vector control transfectants. ARID1A was another candidate RPPA growth-regulatory species identified that approached statistical significance ($P < 0.1$) and showed similar expression profiles between different USP18 shRNA-dependent RPPAs.

A

PTEN Mutation Status	Number of Cases
Not Mutated	514
Deleted	5
Truncation	1
Missense	2

B

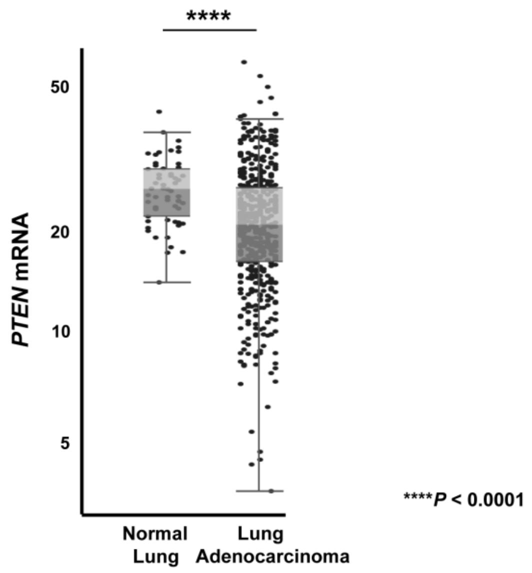

Supplementary Figure S4: *PTEN* mutation patterns did not associate with *PTEN* mRNA expression. (A) Only 2% of TCGA lung adenocarcinoma cases contain alterations in the *PTEN* gene. A table of the number of lung adenocarcinoma cases (*n* = 522) that contain wild-type (*n* = 514) or altered (*n* = 1 truncating mutation, *n* = 2 missense mutations, *n* = 5 deletions) *PTEN* species is shown. **(B)** *PTEN* mRNA was significantly lower in TCGA lung adenocarcinomas (*n* = 517) as compared to the normal lung (*n* = 59).

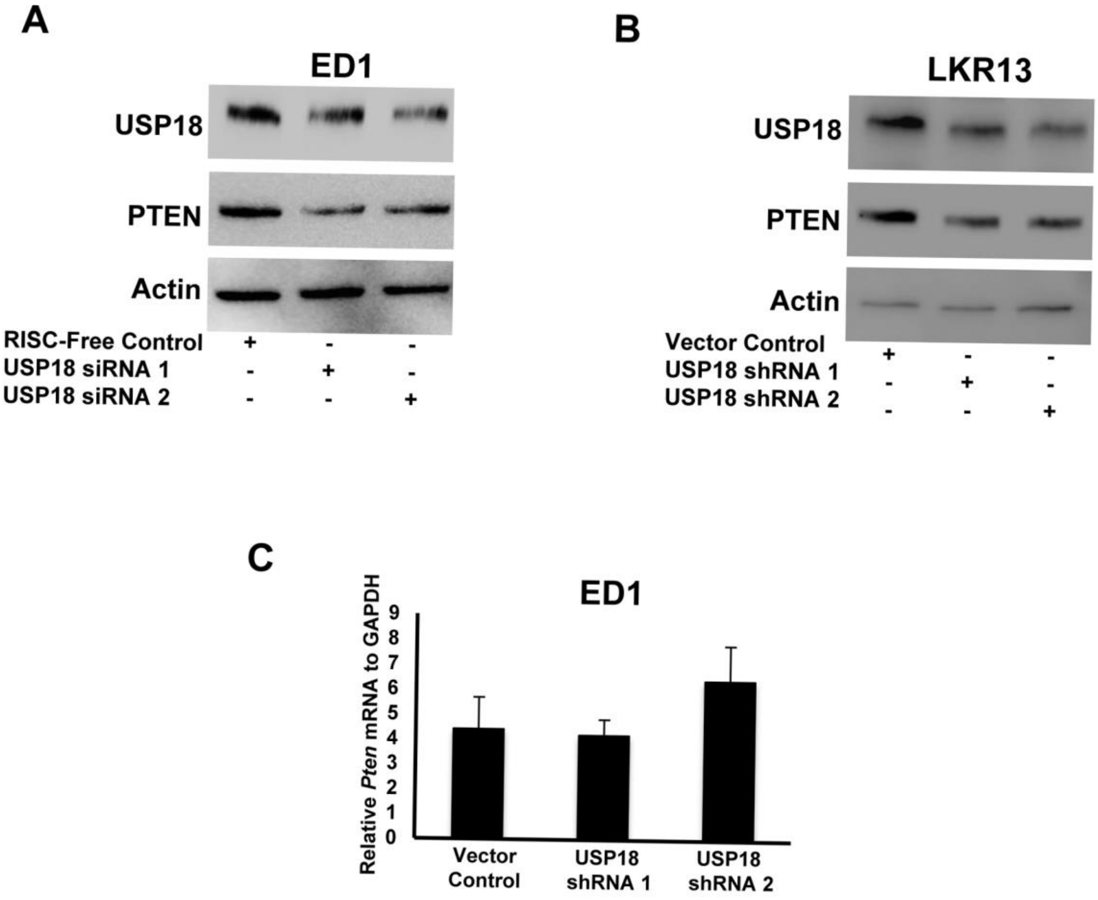

Supplementary Figure S5: USP18 regulated PTEN post-transcriptionally. (A) ED1 murine lung cancer cell lines were independently transfected with a RISC-free control or one of two individual siRNAs against USP18 and **(B)** LKR13 murine lung cancer cells were stably transfected with a vector control or with one of two individual shRNAs against USP18. Immunoblots were probed with individual antibodies recognizing USP18 or PTEN protein. Actin served as a loading control. **(C)** Expression of *Pten* mRNA was analyzed by qRT-PCR assays using a primer set recognizing *Pten*. *Pten* mRNA expression was quantified relative to respective *Gapdh* mRNA and normalized to vector control in ED1 lung cancer cells.

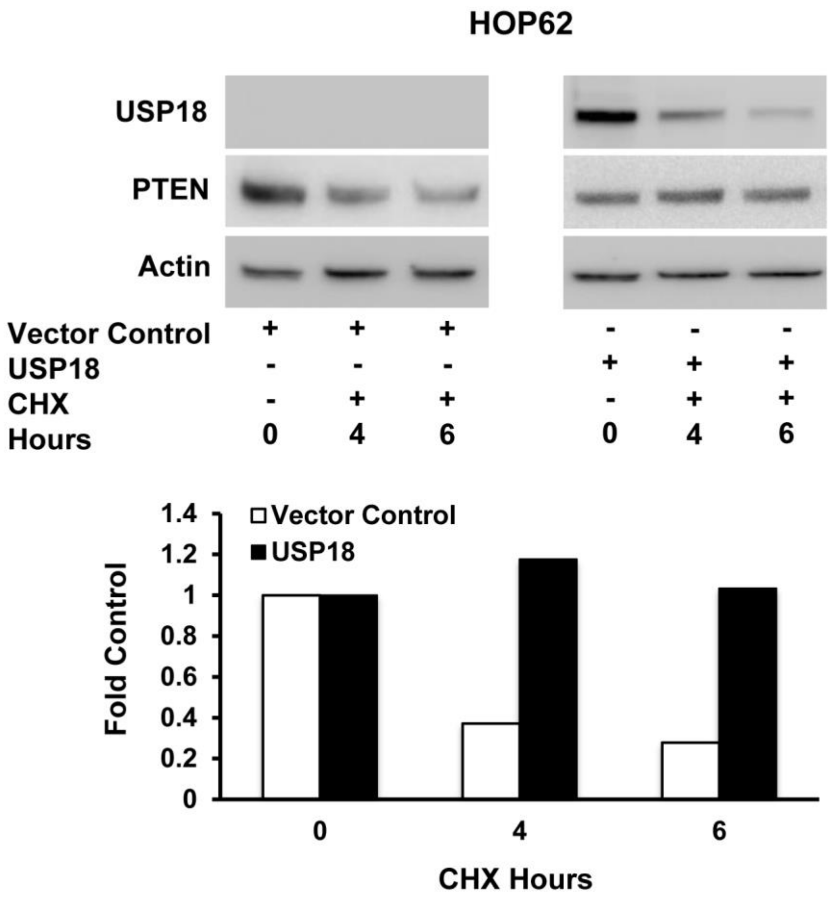

Supplementary Figure S6: Overexpression of USP18 stabilized PTEN protein. HOP62 human lung cancer cells with engineered gain of expression of USP18 or with a vector control were treated with cycloheximide (CHX) for 6 hours. PTEN expression was quantified relative to respective actin expression at indicated time points and normalized to the 0 hour (before CHX treatment) time point. Brief exposure times were used to detect exogenous USP18 protein.

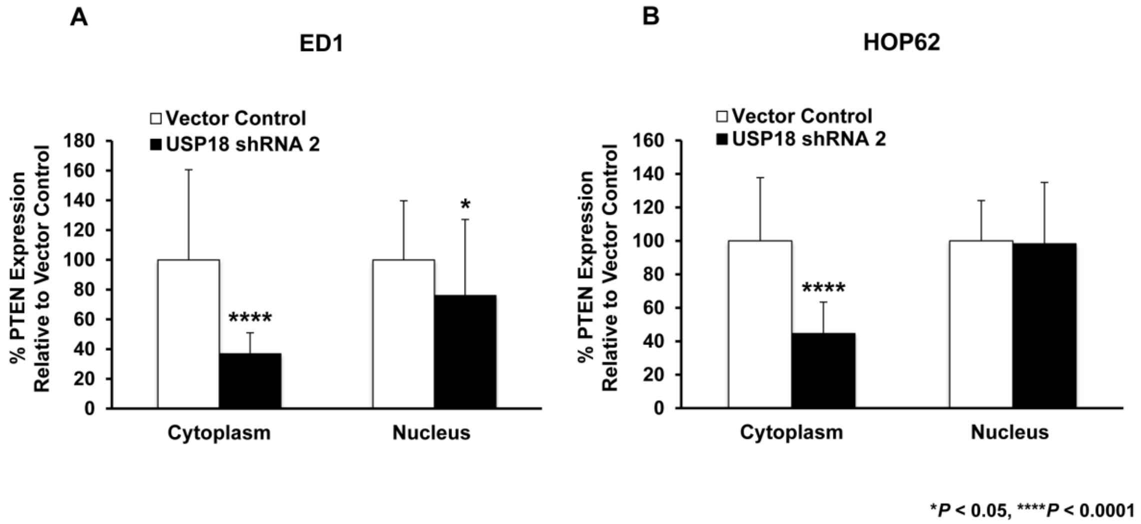

Supplementary Figure S7: Loss of USP18 downregulated cytoplasmic PTEN with minimal effects on nuclear PTEN protein levels. (A) ED1 murine lung cancer cells and **(B)** HOP62 human lung cancer cells stably transfected with a vector control or shRNA against USP18 were individually stained for PTEN, sodium potassium ATPase, and DAPI. Cells were imaged by confocal microscopy. Percent immunostaining of PTEN in the cytoplasm and nucleus of USP18 shRNA-transfected human lung cancer cell lines was quantified at the same intensity as vector control transfected cell lines. To compare differences in PTEN expression, USP18 shRNA expressing lung cancer cell lines were normalized to vector control transfected cell lines. Data are representative of 20 cells per group for each experiment.

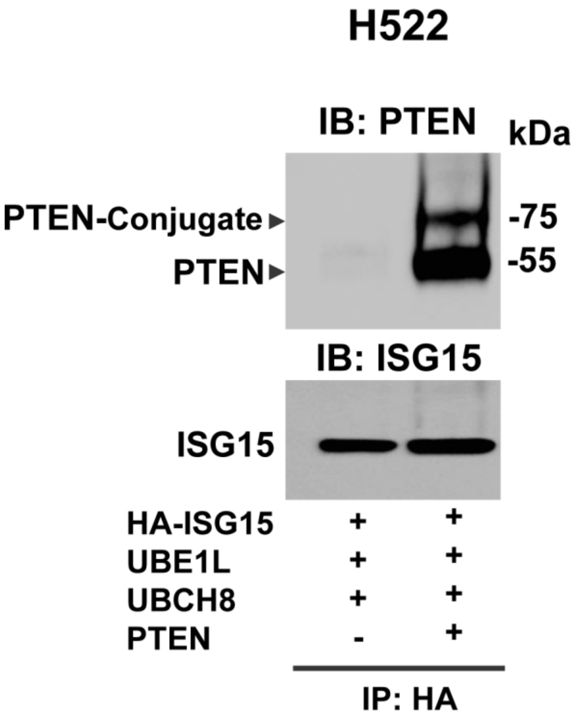

Supplementary Figure S8: PTEN is an ISGylated protein. Immunoprecipitation **(**IP) with an anti-HA antibody followed by immunoblot (IB) with an anti-PTEN antibody revealed ISG15-PTEN complex formation between HA-ISG15 and exogenous PTEN in H522 lung cancer cells.

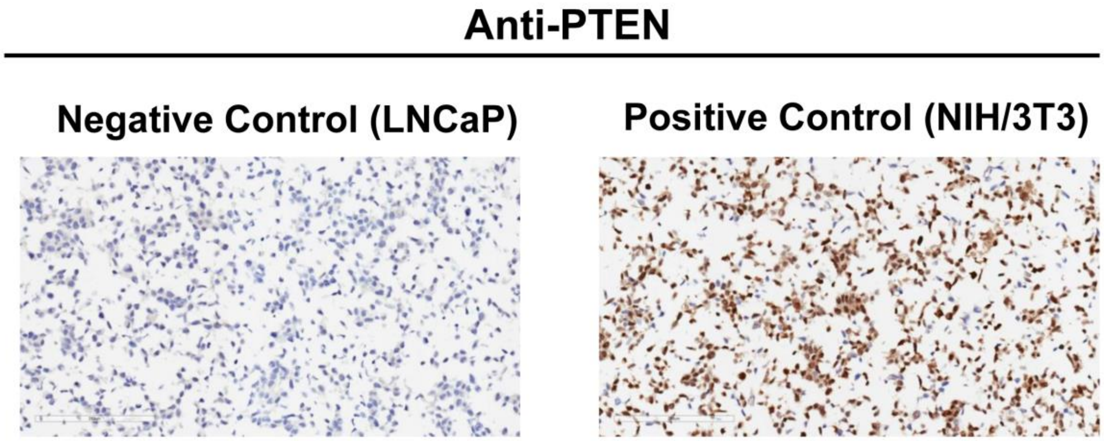

Supplementary Figure S9: Validation of the PTEN-specific antibody for immunohistochemistry studies. The PTEN antibody used for detection was validated using LNCaP (PTEN-null) and NIH/3T3 (PTEN positive) cell lines. For immunohistochemical analyses, cell pellets were fixed in formalin and embedded in paraffin. All magnifications are 20X.

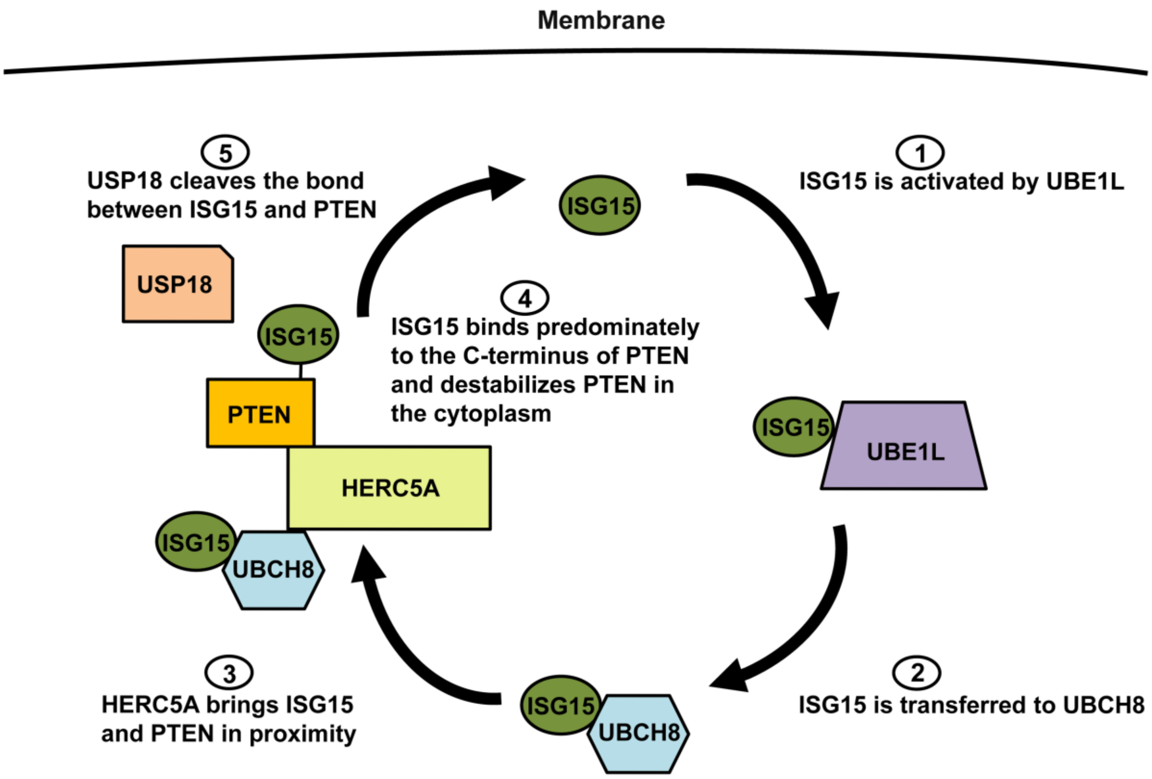

Supplementary Figure S10: PTEN is a target of the ISG15-USP18 pathway. The ISG15 pathway is activated by an **(1)** E1-activating protein (UBE1L), **(2)** an E2-conjugating protein (UBCH8), and **(3)** an E3-ligase protein (commonly HERC5), which facilities ISG15 transfer to the PTEN tumor suppressor protein. Based on findings reported here **(4)** ISG15 conjugated predominately to the C-terminus of PTEN and caused destabilization of PTEN in the cytoplasm. **(5)** USP18, the ISG15-specific deconjugase, cleaves the bond between ISG15 and PTEN and reverses the effects of ISGylation by stabilizing PTEN protein.

Supplementary Table S1: RPPA growth-regulatory proteins identified by loss of USP18 expression in lung cancer cell lines. Expression of the indicated 304 growth-regulatory proteins was analyzed in ED1 and HOP62 lung cancer cell lines stably transfected with a vector control or individual shRNAs that target USP18 for repression. Species in the heat-map of Supplementary Figure S1 are displayed. Results were normalized to linear values of each protein and findings are displayed in the uploaded file.

1	14-3-3 beta	52	Caveolin-1	103	ErbB2/HER2 (phosphoY1248)	154	Lck	205	Pdcd-1L1	256	Smad1
2	14-3-3 epsilon	53	CD171 (L1)	104	ErbB3/HER3	155	LDHA	206	Pdcd4	257	Smad3
3	14-3-3 zeta	54	CD26	105	ErbB3/HER3 (phosphoY1289)	156	LRP6 (phosphoS1490)	207	PDGFR beta	258	Smad4
4	4E-BP1	55	CD29	106	ERCC1	157	MAPK (phosphoT202/Y204)	208	PDHK1	259	Snail
5	4E-BP1 (phosphoS65)	56	CD31	107	ERCC5	158	Mcl 1	209	PDK1	260	SOD1
6	53BP1	57	CD44	108	ERRFI1/MIG6	159	MDM2 (phosphoS166)	210	PDK1 (phosphoS241)	261	SOD2
7	Acetyl CoA Carboxylase (phosphoS79)	58	CD49b	109	Estrogen Receptor	160	MEK1	211	PD-L1	262	Sox2
8	Acetyl CoA Carboxylase 1	59	cdc2 (phosphoY15)	110	Estrogen Receptor alpha (phosphoS118)	161	MEK1 (phosphoS217/S221)	212	PEA-15	263	Src
9	ADAR1	60	cdc25C	111	Ets-1	162	MERIT40 (phosphoS29)	213	PED/PEA-15 (phosphoS116)	264	Src (phosphoY527)
10	Akt	61	CDK1	112	FAK	163	Merlin/NF2	214	PI3 Kinase p110 alpha	265	Src Family (phosphoY416)
11	Akt (phosphoS473)	62	CDKN2A/p16INK4a	113	FAK (phosphoY397)	164	MIF	215	PI3K p110 beta	266	Stat3
12	Akt (phosphoT308)	63	Chk1	114	Fatty Acid Synthase	165	MMP2	216	PI3K p85	267	Stat3 (phosphoY705)
13	AMPK alpha	64	Chk1 (phosphoS296)	115	Fibronectin	166	Mnk1	217	PKA RI alpha	268	Stat5a
14	AMPK alpha (phosphoT172)	65	Chk2	116	FoxM1	167	Monocarboxylic Acid Transporter 4	218	PKC alpha (phosphoS657)	269	Stathmin 1
15	AMPK alpha 2 (phospho S345)	66	Chk2 (phospho T68)	117	FoxO3a	168	MSH6	219	PKC beta II (phospho S660)	270	Syk
16	Androgen Receptor	67	c-Jun (phosphoS73)	118	FoxO3a (phosphoS318/S321)	169	MSI2	220	PKC delta (phosphoS664)	271	Tau
17	Annexin I	68	c-Kit	119	FRA-1	170	mTOR	221	PKM2	272	TAZ
18	Annexin VII	69	Claudin 7	120	G6PD	171	mTOR (phosphoS2448)	222	PLC gamma2 (phosphoY759)	273	TFAM
19	A-Raf	70	c-Met	121	Gab2	172	Myosin heavy chain 11	223	PLK1	274	TIGAR
20	ARID1A	71	c-Met (phosphoY1234/Y1235)	122	GAPDH	173	Myosin IIa (phosphoS1943)	224	PMS2	275	Transferrin Receptor
21	Atg3	72	c-Myc	123	GATA3	174	Myt1	225	PRAS40	276	Transglutaminase II
22	Atg7	73	COG3	124	GCLM	175	NAPSIN A	226	PRAS40 (phosphoT246)	277	TRIM25
23	ATM	74	COL6A1	125	GCN5L2	176	N-Cadherin	227	PREX1	278	TSC1/Hamartin
24	ATM (phospho S1981)	75	Connexin 43	126	Glutamate Dehydrogenase1/2	177	NDRG1 (phospho T346)	228	Progesterone Repector	279	TSC2/Tuberin (phospho T1462)
25	ATR (phosphoS428)	76	Cox2	127	Glutaminase	178	NDUFB4	229	PTEN	280	TTF1
26	ATRX	77	Cox-IV	128	Glycogen Synthase	179	NF-kappaB p65 (phosphoS536)	230	Rab11	281	Tuberin
27	Aurora B/AIM1	78	C-Raf (phosphoS338)	129	Glycogen Synthase (phosphoS641)	180	Notch1	231	Rab25	282	TUFM
28	Axl	79	C-Raf/Raf-1	130	Granzyme B	181	Notch3	232	Rad50	283	Twist
29	B7-H4	80	CREB	131	GSK-3alpha/beta	182	N-Ras	233	Rad51	284	Tyro3
30	Bad (phospho S112)	81	Cyclin B1	132	GSK-3alpha/beta (phospho S21/S9)	183	Oct-4	234	Raptor	285	UBAC1
31	Bak	82	Cyclin D1	133	H2AX (phospho S140)	184	p21	235	Rb	286	Ubiquityl Histone H2B
32	BAP1	83	Cyclin D3	134	Heregulin	185	p27 KIP 1	236	Rb (phosphoS807/S811)	287	UGT1A
33	Bax	84	Cyclin E1	135	HES1	186	p27/KIP 1 (phosphoT198)	237	RBM15	288	ULK1 (phosphoS757)
34	Bcl2	85	Cyclophilin F	136	Hexokinase II	187	p38 MAPK	238	Rheb	289	VASP
35	Bcl2A1	86	Detyrosinated alpha-Tubulin	137	Hif-1 alpha	188	p38 MAPK (phosphoT180/Y182)	239	Rictor	290	VDAC1/Porin
36	Bcl-xL	87	Dimethyl-Histone H3 (Lys4)	138	Histone H3	189	p44/42 MAPK	240	Rictor (phosphoT1135)	291	VEGF Receptor 2

37	Beclin	88	Dimethyl-K9 Histone H3	139	HSP27	190	p53	241	RIP	292	VHL/EPPK1**
38	beta Actin	89	DUSP4/MKP2	140	HSP27 (phosphoS82)	191	p70 S6 Kinase (phosphoT389)	242	Rock-1	293	Vimentin
39	beta Catenin	90	E2F-1	141	HSP70	192	p70/S6K1	243	RPA32	294	Wee1
40	beta Catenin (phosphoT41/S45)	91	E-Cadherin	142	IGF1R (phosphoY1135/Y113)	193	p90RSK (phosphoT573)	244	RPA32 (phosphoS4/S8)	295	WIPI1
41	Bid	92	eEF2	143	IGFBP2	194	PAI-1	245	RSK	296	WIPI2
42	Bim	93	eEF2K	144	IGFRb	195	PAICS	246	S6 (phosphoS235/S236)	297	XBP1
43	BiP/GRP78	94	EGFR	145	INPP4b	196	PAK1	247	S6 (phosphoS240/S244)	298	XPA
44	B-Raf	95	EGFR (phosphoY1173)	146	Insulin Receptor beta	197	PAK4	248	S6 Ribosomal Protein	299	XPF
45	B-Raf (phosphoS445)	96	eIF4E	147	IRF-1	198	PAR	249	SCD	300	XRCC1
46	BRD4	97	eIF4E (phosphoS209)	148	IRS1	199	PARK7/DJ1	250	SDHA	300	XRCC1
47	c-Abl	98	eIF4G	149	Jagged1	200	PARP-1	251	SF2/ASF	301	YAP
48	c-IAP2	99	Elk1 (phosphoS383)	150	Jak2	201	PAX8	252	Shc (phosphoY317)	302	YAP (phosphoS127)
49	Caspase-3 active	100	ENY2	151	JNK/SAPK (phosphoT183/Y185)	202	Paxillin	253	SHP-2 (phosphoY542)	303	YB1 (phosphoS102)
50	Caspase-7 (cleaved D198)	101	Epithelial Membrane Antigen	152	JNK2	203	P-Cadherin	254	SLC1A5	304	ZAP-70
51	Caspase-8	102	ErbB2/HER2	153	LC3A/B	204	PCNA	255	Smac/Diablo		

Expression values of each protein are included in a separate supplementary file.

Data Set:

For Data Set, please see the attached Excel file
